# Adversarial Deep Transfer Learning in Fault Diagnosis: Progress, Challenges, and Future Prospects

**DOI:** 10.3390/s23167263

**Published:** 2023-08-18

**Authors:** Yu Guo, Jundong Zhang, Bin Sun, Yongkang Wang

**Affiliations:** College of Marine Engineering, Dalian Maritime University, Dalian 116026, China; gy79210@dlmu.edu.cn (Y.G.); songhin0414@dlmu.edu.cn (B.S.); wyk_9825@dlmu.edu.cn (Y.W.)

**Keywords:** fault diagnosis, generative adversarial network, transfer learning, domain adaptation, deep transfer learning

## Abstract

Deep Transfer Learning (DTL) signifies a novel paradigm in machine learning, merging the superiorities of deep learning in feature representation with the merits of transfer learning in knowledge transference. This synergistic integration propels DTL to the forefront of research and development within the Intelligent Fault Diagnosis (IFD) sphere. While the early DTL paradigms, reliant on fine-tuning, demonstrated effectiveness, they encountered considerable obstacles in complex domains. In response to these challenges, Adversarial Deep Transfer Learning (ADTL) emerged. This review first categorizes ADTL into non-generative and generative models. The former expands upon traditional DTL, focusing on the efficient transference of features and mapping relationships, while the latter employs technologies such as Generative Adversarial Networks (GANs) to facilitate feature transformation. A thorough examination of the recent advancements of ADTL in the IFD field follows. The review concludes by summarizing the current challenges and future directions for DTL in fault diagnosis, including issues such as data imbalance, negative transfer, and adversarial training stability. Through this cohesive analysis, this review aims to offer valuable insights and guidance for the optimization and implementation of ADTL in real-world industrial scenarios.

## 1. Introduction

In recent years, the diagnosis of machine equipment faults has become increasingly critical, paralleling the continuous advancement and widespread application of industrial technologies [1]. Deep learning, known for its remarkable achievements in image recognition, natural language processing, and more, has consequently attracted significant attention in industrial fault diagnosis. However, traditional deep learning encounters challenges when dealing with limited data in complex industrial equipment and environments, often resulting in suboptimal performance. The conventional assumptions that samples from the training dataset (source domain) and the test dataset (target domain) share the same distribution, and that abundant labeled data are available during training, have further hampered the progression and application of deep learning in IFD. Transfer learning has been introduced to address these limitations by leveraging knowledge from a source domain to assist learning in a target domain. While transfer learning has shown some promising results, practical applications still encounter challenges such as label inconsistency and data distribution mismatch. In response, DTL, an extension of transfer learning, has emerged as a research focus, effectively combining deep learning with transfer strategies to enhance industrial fault diagnosis.

The current literature offers reviews on DTL in fault diagnosis. For instance, Zhao et al. [2] reviewed the IFD problems of unsupervised DTL (UDTL), and studied the transferability of features, the influence of backbones, negative transfer, physical priors, and so on. Li et al. [3] introduced several typical DTL models and the application of DTL in fault feature extraction and fault classification. Qian et al. [4] summarized the application of four deep transfer learning paradigms (instance-based, network-based, mapping-based, and adversarial-based) in intelligent fault diagnosis in recent years. Li et al. [5] summarizes the theory and strategy of the DTL method from the perspective of the algorithm, and puts forward some suggestions for selecting DTL algorithms in practical industrial application. These literature reviews have promoted the development of fault diagnosis in many ways. While these reviews have propelled the field forward, a comprehensive review of fault diagnosis based on ADTL remains missing. A noticeable increase in publications on this specific subject highlights an urgent need for a systematic review. This paper aims to bridge this gap by delving into the advancements in fault diagnosis using ADTL. The majority of early DTL employed fine-tuning techniques to adjust pre-trained model parameters, reducing training costs and enhancing model generalization. However, this approach often fell short when dealing with complex domains and limited datasets. To overcome these shortcomings, ADTL has been increasingly studied and applied. ADTL models can be categorized into generative and non-generative types. Initially, our focus will be on adversarial-based non-generative transfer learning. This extends traditional DTL by integrating adversarial learning mechanisms, intending to establish mapping relationships between the source and target domains for efficient feature transfer. Employing adversarial training helps minimize the distribution discrepancy between the domains, thereby improving generalization performance in industrial fault diagnosis tasks. We will explore non-generative transfer learning both in the context of label consistency, where the source and target domains are identically labeled, and in label inconsistency, where there is a label mismatch. Furthermore, we’ll delve into non-generative transfer learning in complex domains involving multiple sources and targets, with significant differences in data distributions. Subsequently, we will discuss the research progress in generative ADTL for fault diagnosis. Generative models, divided into two types—directly extending data and combining extended data with transfer—enable feature transformation between domains through models such as GANs.

Finally, we will summarize the present challenges and future research status of DTL in TFD. Challenges encompass data imbalance, negative transfer, transferability, adversarial training stability, and more. The proposed future research directions aim to advance DTL application and development in industrial fault diagnosis. This review is intended to offer comprehensive insights to researchers in the field and provide guidance for the optimization and promotion of DTL in real-world industrial applications.

**Figure 1:**
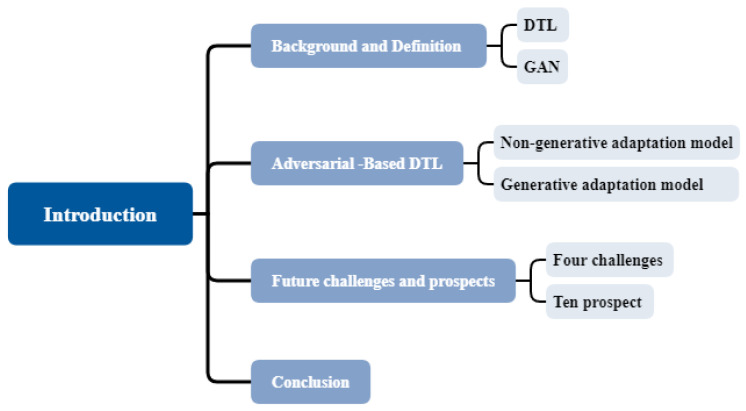
Flow chart showing the overall logic of this literature review.

The remainder of this article is depicted in Figure 1, with Section 2 introducing the basic definition and theoretical background of DTL and GAN. Section 3 classifies and summarizes the latest research progress in IFD based on ADTL, featuring non-generative and generative paradigms. Section 4 highlights four challenges and ten future directions for DTL in IFD. The article concludes in Section 5.

## 2. Background and Definition

### 2.1. Brief Description of the DTL

With references [5,6,7], this article defines domains, tasks, transfer learning, domain adaptation, and DTLs as follows.

Domain contains two components, the feature space X and edge distribution P(X). Write D = {X, P(X)}, where X = {*x* | *x_i_*∈X, i = 1, …, N} is a dataset containing N instances. Usually, different domains are defined according to different feature spaces or different marginal probability distributions. In the mechanical fault diagnosis scenario, different operating conditions, positions and machines can be considered different domains. It should be emphasized that the edge distribution P(X) is usually an implicit function, that is, it is difficult to get an explicit expression.

When given a particular domain D, task T consists of two components, namely, the label space Y and a prediction function *f* (·), denoted as T = {Y, *f* (·)}, where Y = {y|y_i_∈Y, i = 1, …, N} is the label set of the corresponding instance in D. Among them, the prediction function *f* is obtained in the learning process of the algorithm. The prediction function *f* (·) can also be defined as *f* (x) = *P* (y|x), which is expressed as a nonlinear implicit function. It can connect the relationship between input examples and prediction decisions, and is expected to learn from a given dataset. Similarly, different tasks are defined as different label spaces. Different fault categories and types can be regarded as different tasks.

Transfer learning (TL) i.e., given a source domain D^S^ = {X^S^, P^S^(X^S^)} and a source task T^S^ = {Y^S^, *f* ^s^ (·)} and a target domain D^T^ = {X^T^, P^T^ (X^T^)} and a target task T^T^ = {Y^T^, *f* ^T^(·)}, the purpose is to use the transferable knowledge obtained from the source domain and task to learn a better mapping function *f* ^T^(·) for the target task.

Domain adaptation (DA) is an important research direction in the field of transfer learning. The core of this is to reduce the difference in distribution between the two domains, so as to label all the target domain data. The transfer learning in this paper mainly adopts the method of domain adaptation.

Based on the above definition, the definition of deep transfer learning can be expressed as: given a transfer learning task *f*^S→T^(·): X^T^→Y^T^, based on [D^S^, D^T^, T^S^, T^T^], deep transfer learning aims to use a powerful deep learning model, namely, deep neural network, to learn the mapping function *f*^S→T^(·), in which transfer learning technology and the deep learning model can be integrated into a more robust approach to AI.

According to the relationship between source domain and target domain, transfer learning methods are divided into three categories [6,8]: inductive, transductive, and unsupervised. Inductive transfer learning is aimed at the situation wherein the data distribution of source domain and target domain is the same, but the tasks of source domain and target domain are different.

Transductive transfer learning is aimed at the situation wherein the data distribution of source domain and target domain is different, and the tasks of source domain and target domain are the same. Unsupervised transfer learning is aimed at the situation wherein the data distribution of source domain and target domain is different and the tasks of source domain and target domain are different. This classification can also be used for DTL. It should be pointed out that there is no uniform definition of transfer learning at present. Some definitions are still controversial, such as unsupervised transfer learning. Some researchers think that the number of labels in the unsupervised learning target domain should be zero, while some researchers think that there can be a few label data in the target domain. Broadly speaking, as long as we make use of the existing knowledge, models and structures to help us achieve our learning goals on the target data, this process can be called transfer learning. It is worth mentioning that DTL is sometimes called Deep Domain Adaptation. In this article, we do not strictly distinguish between these two concepts. Tan et al. [7] divide DTL methods into Instances-based, Mapping-based, Network-based and Adversarial-based DTL. This article mainly studies Adversarial-based DTL (ADTL).

### 2.2. Theoretical Background of GAN

Goodfellow et al. [9] proposed GAN in 2014, which is a generating framework using minimax games. GAN originated from the generation model, which is used to simulate the samples learned from the raw data distribution. The previous generation model cannot directly reduce the probability distribution distance between the generated data P_g_ and the raw data P_data_. That is, we cannot directly find a generator G* to effectively learn the representation of the raw data:

(1) $$G^{*}=arg\underset{G}{min}\;Div\left(P_{g},P_{data}\right)$$

Inspired by the two-player game, an authentication model called a discriminator is introduced into GAN. As a renewable neural network, discriminator measures the distance between P_g_ and P_data_, which is a great breakthrough in the field of model generation.

**Figure 2:**
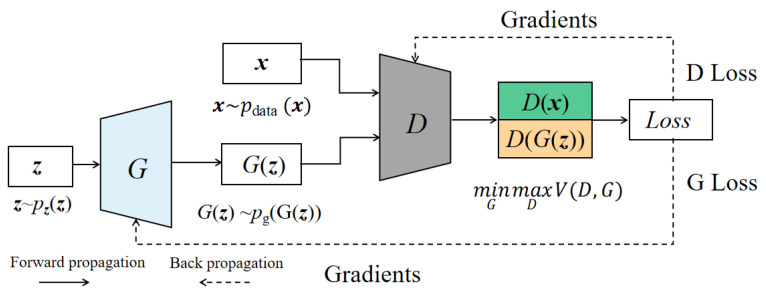
A graphic illustration of the GAN.

Generally speaking, GAN is a generation framework, which aims to match the data distribution of the training dataset. A standard GAN consists of two modules, namely, a generator (G) for learning the potential distribution of training data and a discriminator/critical (D) for distinguishing the sample from the original training dataset. The training process of GANs is shown in Figure 2.

In Figure 2, P_data_ (*x*) is the distribution of real data *x*, p_z_(z) is the distribution of the noise variable *z* (such as standard Gaussian distribution), and generator G (z; *θ_g_*) generates a new sample G(z) distributed as P_g_ according to the randomly input z.

Classifier D (x; *θ_d_*) is like a two-class classifier. Its purpose is to predict whether the input is sample x or G(z). The value function of V(G,D) is

(2) $$\underset{G}{min}\;\underset{D}{max}\;V\left(D,G\right)=E_{x∼p_{\mathrm{data}\;}\left(x\right)}\left[\log D\left(x\right)\right]+E_{x∼p_{z}\left(x\right)}\left[\log\left(1-D\left(G\left(z\right)\right)\right)\right]$$

When training GAN, we usually adopt the strategy of maximum–minimum alternating optimization. On the one hand, the loss of the feature extractor is minimized so that it can generate more realistic samples. On the other hand, the loss of the discriminator is maximized so that it cannot judge whether the given sample comes from real data or generated data.

Specifically, the training strategy fixes one model and updates the parameters of the other model through the stochastic gradient descent (SGD) algorithm.

Here, the parameter update of G is a single-step process, while the parameter update of D can be a single-step process or a multi-step process. In the training process, m noise samples {*z*^(1)^, …, *z*^(m)^} are selected from the raw dataset *P_data_*(x). We select m samples {*x*^(1)^, …, *x*^(m)^} on *P_g_*(***z***) to generate a small batch of samples.

The parameter *θ_d_* of D(x; *θ_d_*) is updated as follows:

(3) $$\nabla_{\theta_{d}}\frac{1}{m}\sum_{i=1}^{m}\;\left[\log D\left(x^{\left(i\right)}\right)+\log\left(1-D\left(G\left(z^{\left(i\right)}\right)\right)\right)\right]$$

The process is repeated several times and the parameter *θ_g_* of G (z; *θ_g_*) is updated as well:

(4) $$\nabla_{\theta_{g}}\frac{1}{m}\sum_{i=1}^{m}\;log\left(1-D\left(G\left(z^{\left(i\right)}\right)\right)\right)$$

We have enough training data to get the best:

(5) $$D^{*}\left(x\right)=\frac{p_{\mathrm{data}\;}\left(x\right)}{p_{\mathrm{data}\;}\left(x\right)+p_{g}\left(x\right)}$$

Through alternate iterations, when *P_g_* = *P_data_*, the global optimization of the minimax game can be achieved theoretically, which means that the new samples generated by G completely conform to the real sample distribution. The detailed description and analysis of GANs can be seen in [9].

In 2017, Arjovsky et al. [10] first proposed Wasserstein GAN (WGAN), which is a great progress of GAN theory. In the field of fault diagnosis, GAN-based data enrichment algorithms have received extensive attention in the small sample problem of fault diagnosis [11,12,13,14,15]. In addition, Guo et al. [16] proposed to generate transfer learning (GTL) to improve the accuracy of the machine intelligent fault diagnosis algorithm under variable working conditions. Liang et al. [17] used GANs and time-frequency imaging technology to propose small sample intelligent fault diagnosis methods, WT-SSGANs. A single and simultaneous troubleshooting learning framework was further studied in [18]. Zhang et al. [19] propose a multi-module generative adversarial network that enhances adaptive decoupling strategies, using adaptive learning methods to update potential vectors. Zheng et al. [20] proposed a MACNN-BiLSTM method based on the multi-scale attention fusion mechanism, which stably generates fault samples of different scales through progressive adversarial training.

## 3. The Research Progress of Adversarial-Based DTL

The essence of GAN is to generate fake data. According to whether synthetic data are generated or not, the ADTL is summarized as generative or non-generative DTL.

### 3.1. Non-Generative Adversarial Adaptation Model

Wei et al. [21] propose a two-stage variable load fault diagnosis method combined with the gradient inversion layer of the adversarial training strategy. It learns the optimal model under new load conditions with labeled and unlabeled data. Jang et al. [22] proposed to introduce an attention mechanism in the adversarial domain adaptation model, extract the features of the attention fault signal, and share spatial information between the discriminator of the feature generator and the hidden layer. Deng et al. [23] proposed a sample-weighted federated adversarial network (SWJAN), which utilizes categorical information to enhance federated domain adaptability for adversarial learning. Ma et al. [24] proposed a collaborative adversarial deep transport model based on convolutional auto-encoder (CADTA). A joint subspace feature identification method including duplex adversarial learning is proposed to improve the identifiability of categorical-level features. In Chai et al. [25], a domain-adaptive (FANDA) method based on a fine-grained adversarial network is proposed. FANDA is characterized by learning by competing with multi-domain discriminators, and realizing the fine-grained alignment of each fault class across two domains. She et al. [26] proposed an adversarial diagnosis method based on the weighted entropy minimization of rotating machinery variable working conditions. Weighted entropy minimization can alleviate model crash problems in domain-adaptive adversarial training, increasing the separation boundaries of categories.

Most of the above studies are based on closed sets (the source domain and the target domain share the same label space) and there are plenty of samples. However, in the actual industrial scene, the working conditions are complex and changeable, and the mechanical equipment is difficult to operate for failure, resulting in the difficulty of obtaining fault data, and the overall data are incomplete.

**Figure 3:**
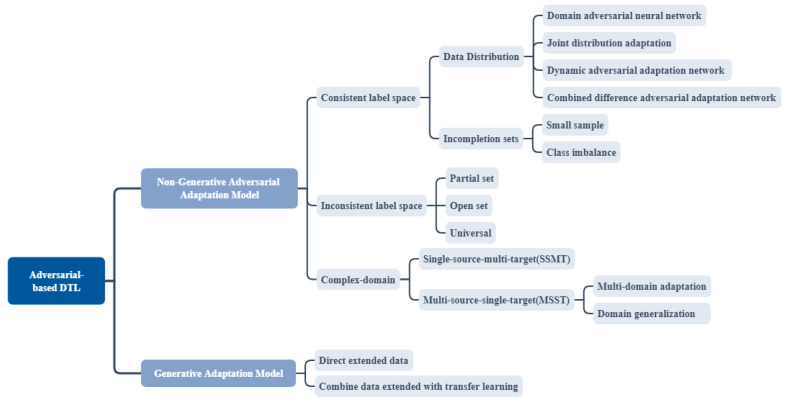
Categorization of ADTL.

**Figure 4:**
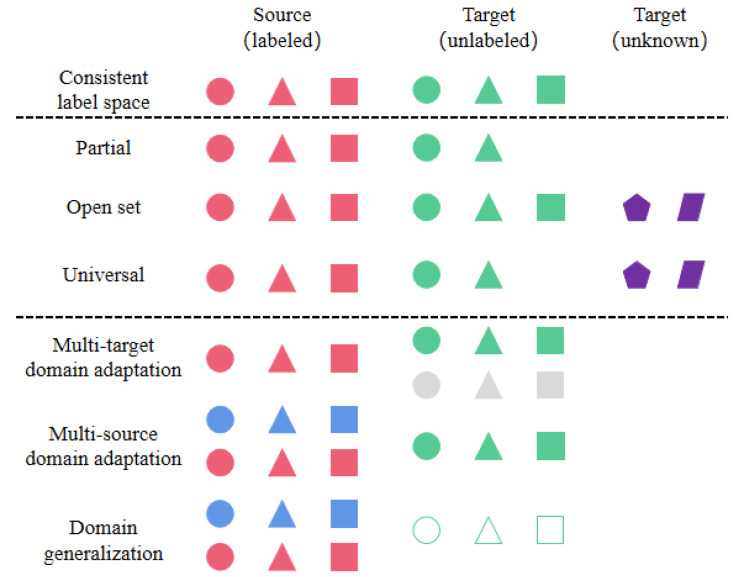
Visualization explanation of different transfer settings. Different colors represent different domains, and hollow shapes indicate that this domain is not involved in training.

In addition, in practical applications, the number of source domains and target domains may not be unique, and multi-domain adaptation also has a very important research prospect. This section introduces the classification of IFD non-generating ADTL from three aspects: consistent label space, inconsistent label space and complex domain. As shown in Figure 3, consistent label space can be divided into two aspects: data distribution and incompletion sets. Inconsistent label space can be divided into partial, open and universal sets. Complex domain can be divided into multi-target domain adaptation, multi-source domain adaptation and domain generalization. It should be noted that the consistent label and inconsistent label also exist in the complex domain. Figure 4 is a visual explanation of the classification method.

#### 3.1.1. Consistent Label Space

##### Data Distribution

Traditional machine learning assumes that the training data and test data of the model follow the same data distribution, while in real applications, the data distributions of the training data and the test data are often different, and the data in the source domain and the target domain usually come from different data distributions, making it difficult to directly achieve good results on the target domain data of the model trained on the source domain. Therefore, how to measure and reduce the distribution difference between the two domains so that the model in the source domain can be better generalized to the target domain becomes the core problem in the field of DTL.

(1) Domain-Adversarial Neural Network (DANN)
  (a) Theoretical Background

Ganin et al. [27,28] first added adversarial mechanisms to the training of neural networks, which the authors refer to as domain-adversarial neural networks (DANN). DANN are defined to solve the problem of different edge distributions between the source domain and target domain. The main idea of DANN is to use the characteristics of GAN in the training process, so that the feature extractor and the domain discriminator are trained against each other, so as to learn the invariant characteristics of the domain.

**Figure 5:**
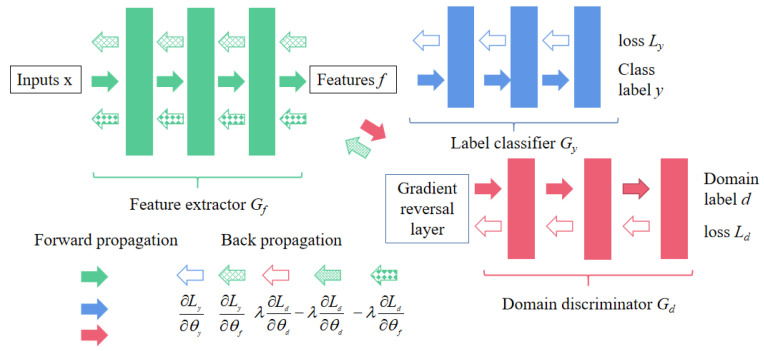
Schematic diagram of DANN.

As shown in Figure 5, DANN consists of the following three parts: feature extractor G_f_, classifier G_y_, and domain discriminator G_d_. G_f_ are used to receive data from the source domain or target domain and extract features; G_y_ is used to receive the extracted features for task classification (it can also be used for other types of downstream tasks); G_d_ is used to judge whether the input feature comes from the source domain or the target domain with the domain label d. The loss of G_d_ is:

(6) $$\begin{array}{c} L_{d}\left(\theta_{f},\theta_{d}\right)=L_{d}\left(G_{d}\left(G_{f}\left(x;\theta_{f}\right);\theta_{d}\right),d_{i}\right) \end{array}$$

where x is the input, d_i_ is the output label, and $d_{i}\in \{0,1\}$, θ_f_ and θ_d_ represent the hyperparameters of G_f_ and G_d_, respectively.

In Ganin et al.’s [27] proof of Equation (6), maximization reduces the degree of h-divergence between the source and target domains as a measure of the two distributions, resulting in the indistinguishability of G_f_ features [29]. Therefore, the training loss L_d_ of the domain classifier is the core of implementing the domain-invariant feature space.

Since the labels of the source domain data are available, the features are sent to G_y_ at the same time, and G_y_ is trained in a supervised way. The loss function of G_y_ is:

(7) $$L_{y}\left(\theta_{f},\theta_{y}\right)=L_{y}\left(G_{y}\left(G_{f}\left(x;\theta_{f}\right);\theta_{y}\right),y_{i}\right)$$

where y_i_ is the class label. θ_y_ is the parameter of G_y_, and G_y_ maps the new feature space to the output of the classification task by minimizing the loss L_y_. In other words, Equation (7) guarantees that the feature space is different. Combining domain and label classifiers, the total loss of DANN can be defined as [27]:

(8) $$E\left(\theta_{f},\theta_{y},\theta_{d}\right)=\frac{1}{n}\sum_{i=1}^{n}\;L_{y}^{i}\left(\theta_{f},\theta_{y}\right)-\lambda \left(\frac{1}{n}\sum_{i=1}^{n}\;L_{d}^{i}\left(\theta_{f},\theta_{d}\right)+\frac{1}{n^{\prime }}\sum_{i=1}^{n^{\prime }}\;L_{d}^{i}\left(\theta_{f},\theta_{d}\right)\right)$$

where n and n’ are the number of samples of source and target, respectively. λ is the weight coefficient of loss.

Corresponding to the training goal of GAN, DANN first optimizes the parameters θ_f_ and θ_y_ of feature extractor G_f_ and classifier G_y_ by minimizing the classification loss and the feature extractor loss.

(9) $$\left({\hat{\theta }}_{f},{\hat{\theta }}_{y}\right)=arg\underset{\theta_{f},\theta_{y}}{min}E\left(\theta_{f},\theta_{y},{\hat{\theta }}_{d}\right)$$

DANN optimizes its parameter θ_d_ by maximizing the loss of domain discriminator G_d_.

(10) $${\hat{\theta }}_{d}=\underset{{\;\theta }_{d}}{\arg max}E\left({\hat{\theta }}_{f},{\hat{\theta }}_{s},\theta_{d}\right)$$

Similar to the training of a GAN, the two steps alternate until the network converges, where ${\hat{\theta }}_{f},{\hat{\theta }}_{s}$ and ${\hat{\theta }}_{d}$ represent the optimal value of saddle point, which can be realized by using the gradient descent optimizer (such as SGD, Adam or RMSProp). The joint optimization of Equations (9) and (10) brings about the minimax training goal of DANN, that is, the feature extractor G_f_ learns the domain-invariant features between the source domain and the target domain as much as possible.

For the convenience of implementation, the author introduces a Gradient Reversal Layer (GRL) into the back-propagation of the network to promote [28] a gradient update. When propagating forward, GRL is an identity map:

(11) $$R_{\lambda }\left(x\right)=x$$

With backpropagation, the gradient is reversed by multiplying by a negative unit (identity matrix I):

(12) $$\frac{dR_{\lambda }\left(x\right)}{dx}=-\lambda I$$

I is the identity matrix. Unlike the two-stage training of GAN [30], GRL can perform synchronous adversarial training on the source and target domain samples, reducing the complexity of the algorithm’s implementation.

The learning goal can be expressed as in Equation (13), and the saddle point can be sought by minimizing the total los $\left({\hat{\theta }}_{f,}{\hat{\theta }}_{s,}{\hat{\theta }}_{d}\right)$ [28].

(13) $$\begin{array}{c} \tilde{E}\left(\theta_{f},\theta_{y},\theta_{d}\right)=\frac{1}{n}\sum_{i=1}^{n}\;L_{y}^{i}\left(G_{y}\left(G_{f}\left(x_{i};\theta_{f}\right);\theta_{y}\right),y_{i}\right)+\\ \frac{1}{n}\sum_{i=1}^{n}\;L_{d}^{i}\left(G_{d}\left(R_{\lambda }\left(G_{f}\left(x_{i};\theta_{f}\right)\right);\theta_{d}\right),d_{i}\right)+\\ \frac{1}{n^{\prime }}\sum_{i=n+1}^{n^{\prime }}\;L_{d}^{i}\left(G_{d}\left(R_{\lambda }\left(G_{f}\left(x_{i};\theta_{f}\right)\right);\theta_{d}\right),d_{i}\right) \end{array}$$

From the point of view of data distribution adaptation, we can find that DANN can be regarded as an adversarial method of edge distribution adaptation. This is because the discriminator receives the overall characteristics of the source domain and the target domain. Tzeng et al. [31] proposed an adversarial discriminative domain adaptation (ADDA). The feature extractor is trained on a sample of the source domain, then both the feature extractor and the domain discriminator are trained through adversarial learning, after which both the feature extractor and the domain discriminator are trained through adversarial learning.

While DANN can effectively adjust the distribution of two domains, there may be bottlenecks in that DANN cannot capture complex multimode structures, and it is difficult to safely adjust domain discriminators. In addition, Zhao et al. [32] have also theoretically demonstrated that it is not enough to reduce the difference in edge distribution between the source domain and the target domain. In this regard, Long et al. [33] proposed a new adversarial-based UDTL model, called CDAN, which aims to reduce the distance between the source and target domains of the conditional probability distribution, thereby completing transfer learning.

Zhu et al. [34] proposed a deep network of conditional probability adaptation that can perform fine-grained feature learning and achieve better results than DANN.

    (b) Applications to IFD

Jiao et al. [35] proposed a double-layer adversarial domain adaptive network (DL-ADAN) for cross-domain fault diagnosis, which consists of two label classifiers for two minimax adversarial games. Jin et al. [36] combined the domain adversarial neural network and the residual network to diagnose bearing faults on unlabeled datasets and improve model performance. Mao et al. [37,38,39] studied early failure detection methods, and the core idea was to optimize DANN to extract domain-invariant features with stronger differentiation. Inspired by DANN, Wang et al. [40] proposed a transmission capsule network based on domain adversarial training. Specifically, the fault characteristics are extracted through wide convolution and multi-scale convolution; fault classification is carried out through the capsule network, and the purpose of enhancing the diagnostic performance of the target domain is achieved through adversarial training. Wang et al. [41] introduced DANN in fault diagnosis, improving the generalization capability of the network in the absence of sufficient labeled data. Zhu et al. [42] argue that heterogeneous data standardization strategies can eliminate differences between different datasets, and proposed a strategy that can guide the selection of DANN hyperparameters. Mao et al. [43] combined DANN with structured correlated information to analyze intrinsic similarities between cross-domain samples. Wu et al. [44] proposed a lightweight domain adversarial neural network (LDANN) in which a lightweight feature extractor is constructed. Di et al. [45] propose a method based on cohesion evaluation and DANN, and unlabeled source domain data are also used for the training of domain classifiers.

(2) Joint Distribution Adaptation (JDA)
  (a) Theoretical Background

Long et al. [46] proposed an adaptive method for joint distribution, the goal of which is to reduce the distance between the joint probability distribution of the source domain and the target domain, so as to complete the transfer learning. In particular, since the joint distribution cannot be directly measured, the joint distribution adaptive method uses the sum of the edge distribution distance and the conditional distribution distance between the source domain and the target domain to approximate the joint distribution distance between them.

    (b) Applications to IFD

Jiao et al. [47] proposed a joint distributed adaptive adversarial network (RJAAN) using residual networks for IFD. Zhao et al. [48] use an improved joint maximum mean difference (IJMMD) method to precisely match feature distributions. Li et al. [49] proposed a deep-transport network (AJDA) with adaptive joint distribution that uses adversarial training with gradient punishment to guide feature generators to provide domain-invariant features between two domains. Yang et al. [50] aligned source and target domains using the Combined Maximum Mean Deviation (JMMD) criterion and the Conditional Domain Adversarial (CDA) learning domain adaptation network based on the deep residual shrinkage network. Zhang et al. [51] proposed a transfer learning method called selective normalized multiscale convolutional adversarial networks. We improve domain alignment by minimizing the difference in the maximum mean of unions in the last multilayer.

(3) Dynamic Adversarial Adaptation Network (DAAN)
  (a) Theoretical Background

Wang et al. [52,53] proposed an adaptive method for dynamic distribution. This method can adaptively adjust the importance of edge and conditional distributions during distribution adaptation according to specific data domains. The dynamic distribution adaptive method dynamically adjusts the distance between two distributions by employing a balance factor μ. Yu et al. [54] made the first attempt to perform dynamic adversarial distribution adaptation for deep adversarial learning, and they proposed a new dynamic adversarial adaptation network (DAAN) to dynamically learn the invariant representation of the domain, while quantitatively assessing the relative importance of global and local domain distributions. Through experiments, it has been proven that there is a mismatch between edge distribution and conditional distribution in the adversarial network. Among them, the adaptive factor is used to dynamically measure the importance of edge distribution and conditional distribution during transfer.

    (b) Applications to IFD

Jiao et al. [55], Tian et al. [56] and Xu et al. [57] proposed an intelligent framework for mechanical fault diagnosis based on adversarial adaptive networks, using adaptive factors to dynamically weigh the relative importance of the two distributions. Wei et al. [58] proposed a dynamic transfer adversarial learning (DTAL) network for handling unsupervised fault diagnosis tasks. The conditional distribution of the local area makes the model independent of training multiple classifiers, reducing the amount of computation of the method. Zhao et al. [59] introduced the attention mechanism into the deep adversarial network, and the attention mechanism determines the weights of different scales, improving the dynamic adjustment performance and adaptive ability of the model. Based on dynamic adaptive thinking, Fan et al. [60] proposed a weighted quantile difference (WQD) measure and integrated it into a deep adversarial learning framework, a method that can effectively learn domain-invariant features to perform different domain adaptation tasks.

(4) Combined Difference Adversarial Adaptation Network (CDAAN)
  (a) Theoretical Background

Li et al. [5] summarized the adversarial DTL compared to feature-based DTL from the mechanism of transfer learning technology to compensate generalization errors in target domain and source domain. The essence of feature-based deep transfer learning is to use the mapping function as a bridge to convert the raw data in the source and target domains from different feature spaces into common potential feature spaces, where differences between domains can be reduced. There are two main research methods. One is to reduce the difference of distribution based on the standard of difference (also known as explicit distance), and the other is to encourage domain confusion by adding domain differentiation architecture through the adversarial mechanism (also known as implicit distance).

There are two main research methods; one is to reduce the difference in distribution based on the criterion of difference (which can also be called explicit distance experimental distance), and the other is to encourage domain confusion (also known as implicit distance) by adding a domain-sensitive architecture through an adversarial mechanism.

Difference-based domain adaptation has been proven to be successful in the field of fault diagnosis. Common metrics include Maximum Mean Discrepancy (MMD) [61], KL divergence [62], multiple kernels MMD (MK-MMD) [63], Wasserstein Distances (WD, also known as Earth-Mover Distance) [10], and Correlation Alignment (CORAL) [64]. But this method is a specific, predefined function based on human priors, for when you do not know which measure to choose. Domain confusion can be encouraged by adding domain-specific architectures through adversarial mechanisms. More and more researchers are studying how to combine the two methods. This section introduces the three metrics of MMD, WD, and CORAL, and summarizes their research progress in fault diagnosis in combination with adversarial mechanisms.

MMD is the most frequently used measure in transfer learning. The MMD measures the distance between two distributions in Reproducing Kernel Hilbert Space (RKHS) [65], and is a nuclear learning method. For two sets of random variables with n_1_ and n_2_ elements, the MMD distance between the two random variables is

(14) $${\mathrm{MMD}}^{2}\left(X,Y\right)={\left\|\frac{1}{n_{1}}\sum_{i=1}^{n_{1}}\phi \left(x_{i}\right)-\frac{1}{n_{2}}\sum_{j=1}^{n_{2}}\phi \left(y_{j}\right)\right\|}_{H}^{2}$$

where ϕ (·) is a mapping, which is used to map the original variable into the RKHS.

WD is a measurement method used to measure the distance between two probability distributions. This distance is defined on a metric space (*M*, *p*), where *p*(x, y) represents the distance function of two instances x and y in the set *M*, such as Euclidean distance. The P-th Wasserstein distance between two probability distributions P and Q can be defined as

(15) $$W_{p}\left(P,Q\right)={\left(\underset{\mu \in \Gamma \left(P,Q\right)}{inf}\int {p\left(x,y\right)}^{p}d\mu \left(x,y\right)\right)}^{1/p}$$

where Γ(P, Q) is all the joint distributions with P and Q as edges in the set *M* × *M*. The famous Kantorovich–Rubinstein theorem indicates that when M is separable, the first Wasserstein distance can be equivalently expressed as an integral probability metric:

(16) $$W_{1}\left(P,Q\right)=\underset{∥f{∥}_{L}⩽1}{sup}E_{x∼P}\left[f\left(x\right)\right]-E_{x∼Q}\left[f\left(x\right)\right]$$

where ${\left\|f\right\|}_{L}=sup\left|f\left(x\right)-f\left(y\right)\right|/p(x,y)$ and $\;{\left\|f\right\|}_{L}\leq 1$; it is also called 1-Lipschitz. Specific details can be found [10].

Sun et al. [64] proposed CORAL, which aligns two domains with second-order features. Assuming that C_s_ and C_t_ are covariance matrices of the source and target domains, respectively, the CORAL method learns a second-order feature transformation A to minimize the feature distance between the source and target domains:

(17) $$\underset{A}{min}{∥A^{T}C_{s}A-C_{t}∥}_{F}^{2}$$

CORAL loss is defined as the second-order statistical feature distance between the source domain and the target domain, where D is the feature dimension of the data:

(18) $$\ell_{CORAL}=\frac{1}{4d^{2}}{∥C_{s}-C_{t}∥}_{F}^{2}$$

Sun et al. [64] calculated the CORAL metric as the loss of a neural network and generated Deep CORAL. It is worth noting that the CORAL method is simple to implement and does not need to specify hyperparameters at all, so it is also convenient to use and achieves good results on specific tasks.

    (b) Applications to IFD

##### Combined MMD(C-MMD)

In refs. [66,67,68,69,70,71], it was shown that MMD assists adversarial domain adaptation to match the distribution of features between different domains. Li et al. [72] used two feature extractors and classifiers, trained using MMD and domain adversarial training, respectively, and used ensemble learning to obtain the final results. Li et al. [73,74] aligned the target domain features with the source domain features by adding MMD in the feature extraction stage. Zhou et al. [75] and Wan et al. [76] used MK-MMD and domain discriminators to adjust the edge and conditional distributions.

##### Combined-WD(C-WD)

Liao et al. [77] sought to train gradient penalty adversarial learning based on both Wasserstein-generative (WGAN-GP) adversarial networks and pseudo-label-based semi-supervised learning, which can generalize the model to fault diagnosis tasks at variable speeds. Li et al. [78] proposed an adaptive semi-supervised framework (C-ASSF) based on current signals. In C-ASSF, Wasserstein generative adversarial networks (WGAN-GP) with gradient penalties are used to extract identifiable features only from normal current signals. He et al. [79] proposed a non-homologous bearing DTL method based on the Wasserstein generative adversarial network (WGAN) and minimum singular value, which uses a domain discrimination network to provide a difference measure to improve domain adaptability.

Inspired by Wasserstein-GAN, Li et al. [80] proposed a cross-domain fault diagnosis adversarial multi-classifier optimization method based on deep learning. Through adversarial training, the over-fitting phenomenon of different classifiers is used to achieve the domain-level adaptive effect, which promotes the extraction of domain-invariant features and the development of cross-domain classifiers. In the literature [81,82,83,84,85,86,87,88,89,90], the domain-invariant features are learned by minimizing the Wasserstein distance between the source and target domain distributions through adversarial training. Zou et al. [91] proposed a fault diagnostic model based on deep convolutional Wasserstein adversarial networks (DCWANs). The model sets variance constraints to overcome the limitation that the decision boundaries between different classes in the target domain are not clear enough. Han et al. [92] introduced mixed distance metrics, including WD and MK-MMD, to minimize the difference between the source and target domains. Liu et al. [93] proposed a transfer learning fault diagnosis model based on deep fully convolutional condition Wasserstein adversarial networks. The proposed domain discrimination module maps the category label conditions to the source domain data through a matrix. Introducing category labels into domain adversarial learning, category information and correlations between categories was studied. Wang et al. [94] proposed a deeply adversarial domain adaptive network (DADAN). They combined the network with the supervised instance-based approach to learn the discriminant characteristics with better intra-class cohesion and inter-class separability. Xu et al. [95] proposed the domain adaptive network model with dual adversarial mechanisms (DAN-DAM), and WD and MMD were used to reduce the difference between the two adversarial mechanisms. Ying et al. [96] proposed an asymmetric adversarial domain adaptive method based on Wasserstein distance. A simplified, lightweight architecture is introduced to enhance generalization and representational capabilities, and reduce computational costs.

##### Combined-CORAL (C-CORAL)

Qin et al. [97] proposed a parameter-sharing adversarial domain adaptive network (PSADAN). The method constructs a shared classifier that unifies the fault classifier and the domain classifier to reduce the complexity of the network structure (the number of hyperparameters), and increases the CORAL loss of adversarial training to enhance domain confusion. Li et al. [98] proposed a deep domain adaptive algorithm (DAACA) based on adversarial thinking and CORAL alignment, which adds deep CORALs to adversarial domain adaptation to reduce the distribution difference between the data from the source domain and the target domain. Li et al. [99] proposed an asymmetric mapping adversarial domain adaptation algorithm (ADA-AMCA) based on CORAL alignment. The model is constrained by deep CORAL to prevent the degradation of learning caused by asymmetric mapping and adversarial learning. Zhang et al. [100] proposed a deep sparse filtering model as an extractor domain adaptive method for fault features, in order to ensure the generalization ability and robustness of the model. Z-score normalization and CORAL, respectively, help to reduce the impacts of features with large variance and reduce the offset between the two domains. Table 1 summarizes the data distribution DTL from three application scenarios; that is, varying working conditions, across different machines and other scenarios.

##### Incompletion Sets

In intelligent mechanical equipment used under variable-load, variable-speed complex working conditions for long-term operation, monitoring system-collected data are mostly repeated and do not contain fault characteristics information. The effective label sample data are small, and the collected training data are unbalanced, that is, the amount of fault data is far lower than the number of health data. The small sample size and unbalanced data are the first problems to be considered in practical applications, and they are also one of the most important problems affecting fault diagnosis.

##### Small Sample

For the small sample problem, there are two solutions: one is to optimize the model to extract the common features of the source domain and the target domain datasets, and the other is to use the generative deep transfer network to generate samples for training. This section introduces the first idea, and the second one will be introduced in Section 3.2.

Han et al. [101] proposed a deep adversarial convolutional neural network (DACNN) that introduces adversarial learning into convolutional neural networks (CNNs) as a regularization method. DACNN can make feature representation more robust with limited training data. Li et al. [102] proposed an intelligent method of partial transfer learning (DA-PTL) based on domain adversarial to solve the problem of the lack of a large number of labeled failure samples in real-world scenarios, and the core of DA-PTL is to assign different weights to samples from different domains. Wu et al. [103] proposed a deep transfer maximum classifier difference method (TMCD) based on a small amount of labeled data. The method first uses a small amount of knowledge in the target domain data to generate a secondary sample, and then adopts an adversarial strategy to introduce two different classifiers to classify the failure type, and the experimental results show that the method is effective in the case of fewer labeled data. Xu et al. [104] proposed a cross-category fault diagnosis method (CFDM) based on less lens learning. The proposed method uses the training example of a mechanical part to achieve the fault classification of different mechanical components. Li et al. [105] proposed a rolling bearing fault diagnosis method based on 1D-CNN and small sample learning model C-WGAN, which can be classified when the training data are extremely limited. Wang et al. [106] proposed a new domain adversarial transfer convolutional neural network DATCCNN. DATCNN uses the domain adversarial training strategy for feature transfer, introduces a conditional adversarial mechanism, improves the joint distribution of features and labels into a random linear combination, and realizes the diagnosis of GIS insulation defects in small samples. Han et al. [107] proposed a framework for dealing with the diagnostic problem of sparse target data transmission. The main idea is to pair the source and target data under the same machine conditions and adapt to the individual domain to alleviate the shortage of target data.

##### Class Imbalance

For class imbalance problems, Guo et al. [108] proposed an adaptive method for decoupling the deep domain. Based on the adversarial domain adaptive model, this method adopts a two-stage training strategy to decouple representation learning and classifier adjustment. Yang et al. [109] proposed a diagnostic model based on adversarial networks, DPTL-Net. As the core of a DPTL network, domain asymmetry factors are automatically learned by training domain discriminators with Wasserstein losses separately, and then used to weight PK-MMD-based distributed adaptive modules. Wu et al. [110] proposed the DTL model (deep Imba-DA). This method uses a cost-sensitive deep classifier to solve the category imbalance problem, using domain adversarial subnet with MMD to minimize the marginal and conditional distributional discrepancy between the source and target domain simultaneously. Kuang et al. [111] proposed a class-unbalanced adversarial transfer learning (CIATL) network with unbalanced data as input. In this framework, class imbalance learning is embedded into the adversarial training process, class separation diagnosis knowledge with unbalanced data is learned, and the two-layer adversarial transfer learning including edge distribution adaptation and conditional distribution adaptation is carried out, after which the domain invariant knowledge is learned. Tan et al. [112] proposed a framework called a deep mixed domain adaptive network (MiDAN) to solve both distribution mismatch and data imbalance. Rebalancing mixture training (ReMix) associated with domain adversarial training was proposed, a technique that introduces a decision boundary to relabel the samples. In addition, the strength and weakness learning framework is used to automatically learn typical characteristics and directly mine the hidden information shared by the source and target domains. Xia et al. [113] proposed a new deep-sensing anti-domain adaptive method (DPADA). A novel perceptual loss is proposed to force the target domain and the source domain to have the same distribution, solving the equilibrium problem in adversarial learning. Table 2 shows the common algorithms for incomplete sets.

#### 3.1.2. Inconsistent Label Space

In the past, most domain adaptations belonged to closed domain adaptation, and closed domain adaptation refers to the domain adaptation problem of the same source domain and target domain label space (both domains contain the same object class). In practice, it is difficult to find a source domain that has the same label space as the target domain, and inconsistent label space is more common. According to the inclusion relationship between label sets, we divide DTL with inconsistent labels into partial set tasks, open set tasks and universal tasks. The closed set target label set is the subspace of the source label set. Open set means that the target label set contains unknown labels. The universal set indicates that the label space relationship is unknown.

##### Partial Set

Wang et al. [114] demonstrated that when there are missing classes in the target training dataset, the direct application of adversarial domain adaptation techniques leads to performance degradation. In order to solve this problem, they proposed a two-phase unilateral joint plan. The proposed method uses the interclass relationship of the source domain to align the distribution of the target domain in one direction to the source domain. Liu et al. [115] proposed a partial adversarial domain adaptive model based on stacked autoencoders (SPADA) to solve the problem of fault diagnosis in the partial domain adaptation environment. Two deep-stack autoencoders are designed to extract representative features from the training data (source domain) and the test data (target domain). Studies [116,117,118,119,120,121] use weighted or quasi weighted adversarial networks to solve the partial transfer learning problem. Deng et al. [122] proposed an adversarial network based on two-layer attention (DA-GAN). A two-tiered attention mechanism is designed to facilitate positive transfer and reduce the negative impact of irrelevant source data. Mao et al. [123] proposed a partial transfer ensemble learning framework (PT-ELF). A specific integration strategy is designed to combine a weak global classifier and two partial domain adaptive classifiers to produce the final diagnostic results. Qian et al. [124] proposed a new multi-scale weight selection adversarial network (MWSAN) to enhance the effect of some DA; MWSAN is mainly composed of a multiscale domain adversarial network (MDAN) and a multi-scale weight selection mechanism. In order to suppress the overfitting of the labeled source domain and enhance the local DA, an MDAN containing a multi-classifier is proposed. Guo et al. [125] designed a multi-scale and multi-view domain adversarial network (MMDAN) method to solve cross-condition and partial set fault diagnosis tasks.

##### Open Set

Zhang et al. [126] proposed an open-set domain adaptive method based on deep learning. The introduction of adversarial learning extracts generalized features, and an instance-level weighting mechanism is proposed to reflect the similarity of known health status test samples. Zhao et al. [127] proposed a new type of open set domain adaptation network based on dual adversarial learning. A secondary domain discriminator is used to assign similarity weights to a single target sample to distinguish between known and unknown failure modes. Zhu et al. [128] proposed an ANMAC model. Before adversarial learning, the model underwent a weighting scheme that evaluates labels and domain information, providing distinguishable weights for known and unknown target instances.

Inspired by the idea of open set domain adaptation, Li et al. proposed a global–local dynamic countermeasure network [129], a deep adversarial network based on a stacked autoencoder [130], a deep adversarial transfer learning network (DATLN) [131] and a two-stage transfer adversarial network (TSTAN) [132]. The four methods are applied to new fault diagnosis, and all have achieved good diagnosis results.

##### Universal

Aiming at the universal domain adaptation problem without prior knowledge, Chen et al. [133] proposed a fault diagnosis method based on the transferability quantification TWUAN. According to the data distribution in different working environments, TWUAN embeds auxiliary domain discriminators and auxiliary classifiers in the traditional adversarial domain adaptive model, and designs class-level weights for source and target samples, respectively. Yu et al. [134] proposed a model called a bilateral weighted adversarial network (BWAN) that classifies samples based on the output of the deep model and rejects samples of unknown classes through an extreme-value theory model. Zhang et al. [135] proposed a hybrid selection adaptive method based on the weighting mechanism of source classes and target instances. Using additional outlier identifiers, the method can automatically identify unknown failure modes without knowing the target label set.

Li et al. [136] have developed a fault diagnosis framework (ADGN) for unknown operating environments. ADGN can diagnose failures in unknown operating environments and use only one fully labeled domain in training. Table 3 shows common algorithms for inconsistent label space.

#### 3.1.3. Complex Domain

Complex domain adaptation is used to solve the problem of domain adaptation with multiple target domains or multiple source domains. In the classical domain adaptation problem, the target domain samples are sampled from the same distribution by default, but this is a simplification of the actual situation. In practice, it is more likely to encounter the situation of simultaneous transfer from the source domain to multiple target domains with different distributions. Even in some cases, the target domain samples are unavailable, so only the models with good generalization performance can be trained from the source domain to meet the task requirements in the target domain. This kind of problem is called a complex domain adaptation problem.

In this section, the problem of domain adaptation in the complex domain is divided into single-source–multi-target (SSMT) and multi-source–single-target (MSST), where MSST includes multi-domain adaptation (using target data in training phase) and domain generalization (not using target data in training phase).

##### Single-Source-Multi-Target (SSMT)

Li et al. [137] proposed an adversarial multi-domain adaptive fault diagnosis method (AMDA), which uses single-source domain knowledge to achieve multi-target domain fault diagnosis. Deng et al. [138] proposed a new correlation regularization conditional adversarial adaptation network (CRCAA), which reduces negative transfer near the decision boundary by establishing a correlation regularization mechanism that uses sample correlation to guide distribution alignment. Ragab et al. [139] proposed a deep learning architecture for adaptive adaptation in adversarial unsupervised domains to solve SSMT problems and exposed source code and data.

##### Multi-Source–Single-Target (MSST)

(1) Multi-domain adaptation

Zhao et al. [140] use domain adversarial networks to solve multi-source transfer learning problems. Wei et al. [141] proposed a multi-source adaptation framework for learning features from the original vibration signal that have fault recognition capabilities but unchanged operating conditions. Depending on the similarity of the distribution to the target case, different known cases are assigned different weights. Xu et al. [142] proposed an Intelligent Fault Diagnosis System (IFDS) with a multi-source unsupervised domain adaptive network that can accommodate single-source or multi-source domains. This method considers the differences between sources, and uses source domain data and a small amount of unlabeled target domain data to mine the feature information contained in the data. Si et al. [143] proposed a multi-source domain adaptation (MSDA) strategy. Multi-order moment matching strategy is used to extract generalized knowledge from multiple domains. Chai et al. [144] proposed a multi-source adaptive diagnosis network (MADN). In MADN, SAE is preprocessed to extract a high-level representation from the process data. They utilize multiple domain discriminators to ensure that the learning characteristics within each domain are transferable. Rezaeianjouybari et al. [145] propose feature-level and task-specific distribution alignment multi-source domain adaptation (FTD-MSDA), which transfers knowledge from multiple tagged source domains to a single unlabeled target domain by reducing differences in feature distribution between the target domain and each source domain. Huang et al. [146] proposed an unsupervised fault diagnosis method MDAAN. This involves deep feature extraction and fusion using dense convolutional and fused convolutions. The network incorporates multi-sensor vibration information and classified label information. Zhu et al. [147] proposed a TL method based on multi-source domain adaptation. The method learns the strategy through a multi-faceted approach without the need to select the optimal combination rule for multiple sources.

Aiming at the multi-source domain adaptation problem, Zhang et al. [148] proposed a new adversarial domain adaptation with a classifier alignment (ADACL) method, which uses adversarial learning network structures to perform multi-source domain adaptive tasks, and realizes information sharing between multi-source domains and target domains. Feng et al. [149] proposed a global–local multi-source fault diagnosis method based on class transfer, which realizes multi-source DA from the domain and class levels by locally optimizing the WD of the classifier and the global accumulation of high-order multi-source moments, and adopts an adaptive weighting strategy to ensure the reliability of the results. Li et al. [150] proposed a method based on deep transfer learning. This method considers different levels of fault severity, and when the test data are polluted by additional noise, the proposed transfer learning method can also significantly improve the diagnostic performance. Chai et al. [151] proposed a fault diagnosis method multisource-refined transfer network (MRTN) in the case of inconsistent domains and categories. They refine the classification and adaptation of faults, and align the category distribution within each domain. The negative transfer defect caused by the traditional forced alignment of global domains is avoided. Li et al. [152] proposed a mechanical fault diagnosis method based on deep learning. Distance metric learning is used to enhance model robustness to class-to-class severability and intra-class compactness.

(2) Domain generalization

Chen et al. [153] proposed a new ADIG fault diagnosis framework that, through adversarial training, learns domain invariant and fault-related knowledge from multiple domains. In addition, a customized strategy of feature normalization and adaptive weighting is proposed to improve diagnostic performance. Han et al. [154] proposed IEDGNet, a domain-based hybrid diagnostic network. The main idea is to regularize the deep network discriminant structure with internal and external generalization goals, so that the diagnostic model can learn robust features and generalize to the invisible domain. External domain-level regularization is achieved through adversarial training, further reducing the risk of overfitting. Zhang et al. [155] proposed a conditional adversarial domain generalization method based on a single discriminator, which greatly saved computing resources compared with the traditional conditional adversarial domain generalization. Huang et al. [156] proposed a deep adversarial capsule network DACN, embedding a multi-domain generalization method into intelligent compound fault diagnosis. DACN constructs a decoupling classifier by superimposing capsule layers, and achieve domain generalization by introducing adversarial training to align the feature distribution of multiple domains. Table 4 summarizes the common algorithms for complex domains.

### 3.2. Generative Adversarial Adaptation Model

Generative adversarial adaptation models generally have generators and discriminators, rather than feature extractors in non-generative models. Therefore, the generative model can learn the joint distribution of data from two domains, or transform the data from the source domain to the target domain to achieve domain adaptation.

Generative adaptation models are primarily generated by directly using GANs and their variants to generate new data similar to the real data in the target domain, such as frequency domain data [157,158] and time–frequency domain data. [159,160]. With the help of available source data, and then using these generated and real data to train an additional deep model, a reliable diagnosis result can be achieved. It should be emphasized here that data generation is not the ultimate goal of learning, but the ultimate goal is to achieve better transfer learning effect.

Shao et al. [161], Guo et al. [162], Shi et al. [163] and Wu et al. [164] added an auxiliary classifier to GAN instead of training additional classifiers, so as to make full use of label information and achieve higher diagnostic accuracy with fewer training data. Peng et al. [165] optimized the discriminator by enhancing the generative adversarial network and adversarial mechanism, and used parameter transfer learning (PTL) to solve the problem of fault diagnosis with only a small number of label samples. Li et al. [166] proposed a method of fusing convolution to generate an adversarial encoder (fCGAE), creating a fault detection model only from normal data. In order to match the difference in the probability distribution of the generated data, Zhu et al. [167] proposed the famous CycleGAN, which first transforms the data of the source domain into the target domain through one set of mapping, and then through another map, the source domain data mapped to the target domain are mapped back to the source domain space. This process trains by measuring the difference between the source domain data and the source domain data being mapped back.

If we use F and G to represent the mapping function from source domain to target domain and from target domain to source domain, the training goal of CycleGAN can be represented as:

(19) $$L_{cyc}=E_{{x\sim P}_{data}\left(x\right)}\left[{\left\|F\left(G\left(x\right)\right)-x\right\|}_{1}\right]+E_{{y\sim P}_{data}\left(y\right)}\left[{\left\|F\left(G\left(y\right)\right)-y\right\|}_{1}\right]$$

Xie et al. [168] proposed a cyclic consistency GAN for bearing fault diagnosis under different operating conditions, using existing working condition data to generate target working condition data to solve cross-domain fault diagnosis problems. Inspired by StyleGAN, Wang et al. [169] proposed a transfer fault diagnosis model based on the adversarial generation model, which generates the sample characteristics of the source domain in the feature space into samples that conform to the distribution of the target domain through incremental learning, thereby improving the imbalance of the target domain categories. Jiao et al. [170] proposed cyclic consistent adversarial adaptive networks (CAAN), designing a cycle-consistent generative adversarial constraint to ensure sufficient feature similarity between the source and target domains after adaptation. Zhao et al. [171] proposed a cross-condition data supplemental method for cyclic GAN (CycleGAN) and dynamic models, which can use limited available data to approximate the missing parts of existing data for diagnosis in the target domain. Liu et al. [172] proposed a transfer learning method based on conditional variational GAN (CVAE-GAN), using improved CVAE-GAN to generate missing data under other operating conditions. Zhu et al. [173] proposed IDAL, a method used to solve the problem of industrial fault diagnosis in the unbalanced domain. This method takes into account small samples and domain-to-domain imbalance datasets to achieve automatic feature extraction. Li et al. [174] proposed an adversarial domain adaptation method based on knowledge mapping (KMDA), and the visualization results show that the model extracted the invariant characteristics of the domain and realized the knowledge mapping diagnosis. Apart from direct data generation, some works also consider directly integrating data generation with the transfer learning process. Sankaranarayanan et al. [175] proposed a method called “Generate to Adapt” for domain adaptation using generated data, the source domain, and target data to learn domain-invariant features through adversarial training. On the other hand, Xu et al. [176] introduced the use of Mixup, a data augmentation technique, into transfer learning to learn shared features between the source and target domain data. Currently, there is limited research that combines data generation with the process of transfer learning. Table 5 summarizes the research progress of generative transfer learning. However, a special challenge faced by the generative domain adaptation model is that it is difficult to quantitatively evaluate the quality of generated data with valid metrics.

### 3.3. Summary

The third section of this review presents a thorough evaluation of the research progress within ADTL. This is initiated by examining non-generative adversarial adaptation models and highlighting the significant roles they play in a consistent label space. The studied models include DANN, JDA, DAAN, and CDAAN. Each was scrutinized for its theoretical background and applicability within the IFD framework. The exploration also pinpointed obstacles related to data distribution such as incompletion sets, small sample sizes, and class imbalance, which pose challenges to the implementation and efficiency of the models. Shifting the focus to inconsistent label spaces, the review navigated through the intricacies of partial sets, open sets, and universal sets. It further delved into the complex domain issues like SSMT and MSST, accompanied by an analysis of multi-domain adaptation and domain generalization. The generative adversarial adaptation model was explored, which can be divided into two models: direct extended data and extended data combined with transfer. To summarize, this section provides a comprehensive understanding of the state-of-the-art in adversarial-based DTL and how it is carving its path within the domain of IFD, effectively addressing diverse challenges that arise in the practical application of these models.

## 4. Challenges and Prospects of DTL in Industrial Fault Diagnosis

**Figure 6:**
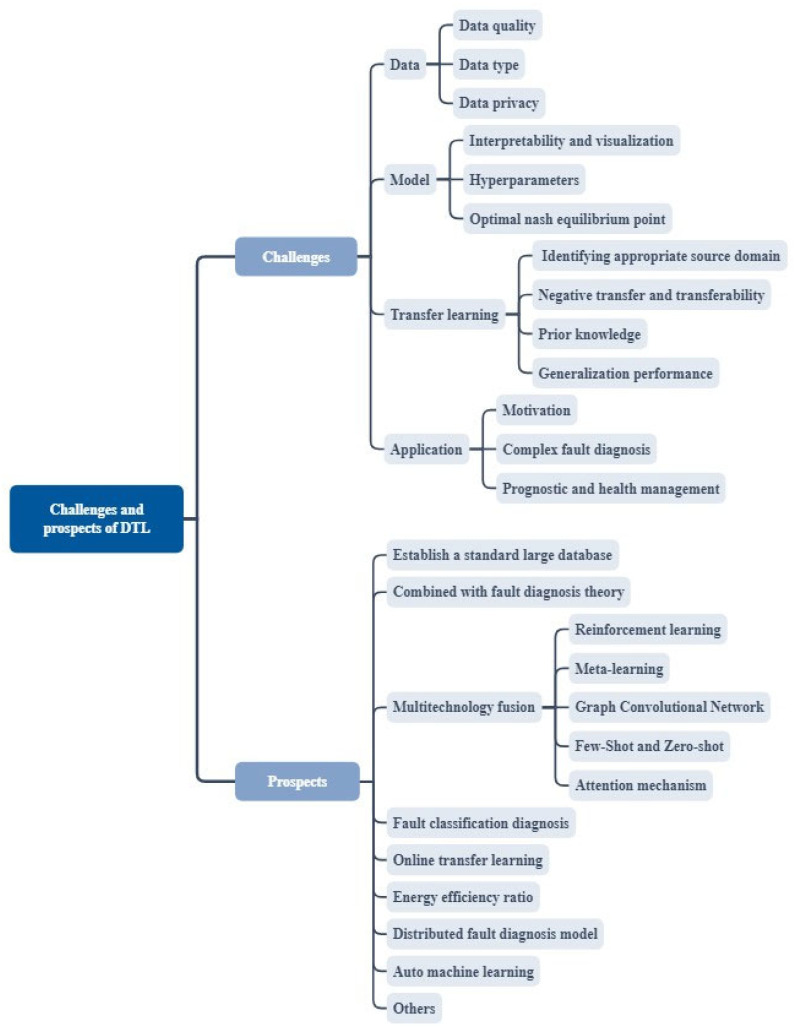
Process diagram for the challenges and prospects of IFD.

Researchers have reviewed the progress and challenges of artificial intelligence and machine learning [177], as shown in Figure 6, and this study identifies several challenges and future research directions related to deep transfer learning in the field of fault diagnosis.

### 4.1. The Challenges of DTL Methods for Fault Diagnosis

**Figure 7:**
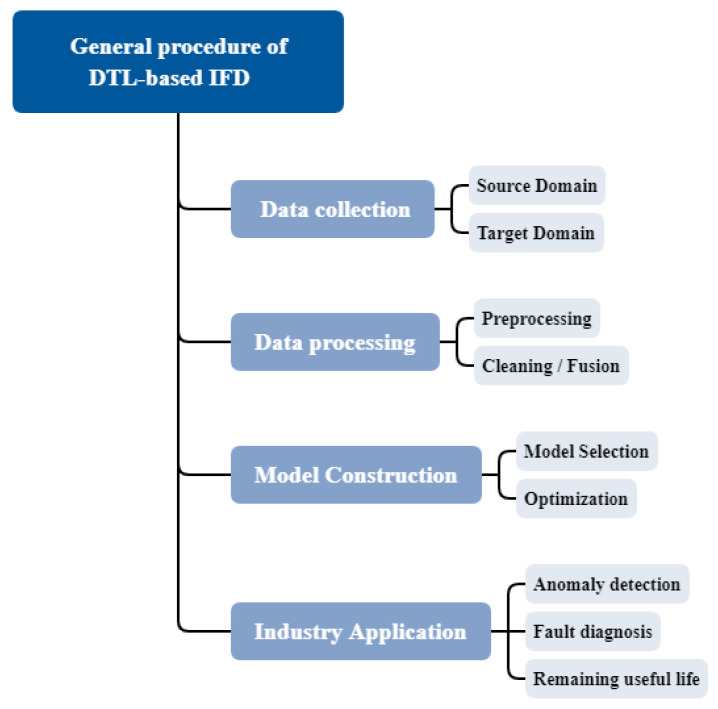
General procedure of DTL-based IFD.

As shown in Figure 7, the general process of IFD based on DTL includes four key steps. After completing these four steps, IFD can be realized in practical application. But there are still many challenges in practical applications.

#### 4.1.1. Data

##### Data Quality

Data are the only information source based on IFD, so the quality of data determines the detection ability and generalization ability of the model. The data signals collected in the actual industrial system are full of many uncertain factors, and there is redundant information in the process data, including some repetitive data and noise interference, which seriously affect the characteristic representation of the data in the model. The literature [178,179,180] proposes a solution to the problem of the strong interference of signals in fault diagnosis to mitigate the negative effects of noise. However, excessive denoising or insufficient denoising can distort the original signal, reducing fault diagnosis efficiency and even accuracy. In the actual industry, the uncertainty of noise level and other factors directly determine the results of fault diagnosis, which still requires attention.

With the development of information technology, massive normal operation data and small sample fault state data are a typical feature of industrial big data. By establishing a dynamic simulation model, a simulation training dataset with sufficient samples and rich label categories can be obtained. Diagnostic knowledge is obtained from simulation data to achieve fault diagnosis in machinery and equipment [181,182,183,184]. In fault diagnosis, there is also an extreme case wherein the fault data sample is missing, that is, there is no fault data in the source and target domains, and the digital twin technology can be considered to complete the fault diagnosis transfer learning [185]. Digital twin technology can be employed through the numerical simulation method to establish a digital twin model of the device, and virtual experiments can obtain fault data that do not exist in practice, through the adversarial domain transfer method, to narrow the domain difference between the simulation signal and the actual sensor signal, such that the fault diagnosis model trained on the virtual entity (digital twin) can be used for the fault diagnosis problem of the physical entity, enabling it to solve the problem according to which the physical entity only has health status data.

##### Data Type

Most of the existing fault diagnosis studies mainly focus on single-channel signals dominated by vibration signals. This is because the vibration signal can be collected by the acceleration sensor attached to the surface of the component, which is sensitive to the impact caused by structural damage, such as gear fracture, bearing outer ring crack, etc. For some non-structural faults, such as oil shortage in the gearbox, the vibration signal is insensitive to them. These failures can also lead to serious consequences and should not be ignored. The literature [186,187] overcomes the limitations of a single vibration signal and utilizes a fusion module to learn shared features. Yu et al. [188] proposed a DTL algorithm JFLAN, which can learn effective features from the graph and solve the problem of the small sample and non-stationary generalization error in semiconductor processes. In addition, there are few studies on uncertainty data. Zaitseva et al. [189] presents a new method for constructing structure functions based on initial uncertain data. The method uses fuzzy decision trees (FDT) to transform the initial uncertain data related to a real system into precisely defined system structure functions.

In industrial scenes, in order to comprehensively monitor the running state of integrated equipment, various types of sensors may be used to collect different signals, such as sound, vibration, voltage, temperature, oil, acoustics and optics. Therefore, multi-source heterogeneous data are also very common, such as one-dimensional signals (vibration signals, displacement signals, current signals, acoustic emission signals) and two-dimensional signals (thermography, time-frequency diagram). It is very important to extract effective features from these multi-source heterogeneous signals for equipment maintenance. At present, few studies pay attention to the transfer learning of this kind of multi-source heterogeneous data in IFD. Therefore, the heterogeneous transfer learning between multi-sensors will be one of the hot topics in the future.

In the field of mechanical fault diagnosis, since the newly acquired data in the operation of actual mechanical equipment are usually unlabeled, unsupervised DTLs are currently the hot topic of research. The fault diagnosis technology based on unsupervised ADTL is studied [190,191,192,193,194,195,196]. Some researchers believe that the absence of any mark in the target domain will cause unnecessary difficulties for some practical cases. When the actual equipment runs for a period of time, it may be a more reasonable choice to study the weak supervised domain adaptation (including semi-supervised learning and unsupervised learning) [194].

##### Data Privacy

In industrial applications, it is often difficult for individual users to collect enough high-quality data due to cost constraints, while industrial users usually have potential conflicts of interest and it is difficult to share their data directly with each other. This hinders the development and application of intelligent fault diagnosis technology. The question, then, is how to make use of the data while ensuring data privacy? Federated learning provides a good solution for the collaborative modeling of data privacy. Zhang et al. [197] proposed a joint transfer learning method for fault diagnosis. The federated initialization phase is introduced to maintain similar data structures in distributed feature extraction, and the federated communication phase is further realized for deep adversarial learning. The limitation of their study is the assumption that all machine health sets are the same, and that cases where the health state sets are different deserve further study. Sun et al. [198] proposed a swarm learning (SL) framework that combines adversarial domain networks with convolutional neural networks (CNNs) to protect data privacy. Zhao et al. [199] proposed a federated multi-source domain adaptation method with data privacy for mechanical fault diagnosis. A federated feature alignment idea is introduced to distill common and similar features of all source and target domains. Zhang et al. [200] presented a federated transfer learning method for mechanical fault diagnosis. In the case that the data of different clients cannot communicate, the prior distribution is proposed to indirectly bridge the domain gap. In the industrial scene, federal learning still has some problems, such as high infrastructure cost and low acceptance by enterprises.

#### 4.1.2. Model

##### Interpretability and Visualization

Although the IFD method based on DTL has made amazing achievements in mechanical fault diagnosis and prediction, its acknowledged limitation is that, similar to deep learning, DTL has a “black box” stage, and its application is mostly in the mode of “inputting data, observing results and adjusting parameters”. The DTL multi-hidden-layer network structure has no scientific and unified theory to explain its internal principle, and there is no scientific reason and rigorous design process for its application in the field of fault diagnosis and prediction.

For the “black box” structure of the DTL model and the platform for building the internal operation mechanism of the visual network, fault diagnosis based on the DTL will be traceable, which is more convenient for the optimization and adjustment of the diagnostic model, and it is believed that it will also promote the research of the internal computer system of DTL learning, enhance the theoretical support, and improve the diagnostic performance of the algorithm.

At present, some visualization methods have been proposed, such as t-SNE diagrams for high-dimensional data visualization [201], and the visualization of the activation generated by each layer of deep neural networks through regular optimization [202]. Some researchers have studied the interpretability of deep networks and achieved some results. For example, the mechanism of conceptual dialects (CW) was introduced to understand the process of layer-by-layer learning of networks [203]. However, the interpretability of the model is still a question worth investigating.

##### Hyperparameters

Although the DTL method has achieved good application results in fault diagnosis, its complex structural parameters need to be intelligently optimized, and parameter selection affects the accuracy of fault sign extraction.

At present, there is no systematic theoretical system that can be used to guide the adjustment of DTL parameters. The adjustment of related parameters often needs to be selected according to actual experience, or the random initialization of parameters through initialization algorithms, or the adjustment of parameters in experiments and continuous attempts. Therefore, the dynamic optimization and adjustment of model parameters during the model-building process will also be a major challenge for its development.

Some researchers have made relevant research attempts on this topic, such as transforming the super-parameter search problem into an optimization problem, and using the genetic algorithm and particle swarm optimization algorithm to get the optimal solution. However, at present, the research in this area is not complete, and more in-deep discussions are needed [204,205]. In the process of super-parameter selection, we can consider the background of the fault diagnosis field and the characteristics of objects, and refer to other structures and super-parameter settings of prediction models in this field, before making adaptive selections and adjustments, so as to improve the rationality of super-parameter selection. In the future, automatic machine learning may be an effective way to solve such problems [206].

##### Optimal Nash Equilibrium Point

The adversarial-based domain adaptation model is trained by the game training strategy, and the optimal Nash equilibrium point needs to be reached between domain generator and domain discriminator to ensure that the model can screen and generate high-quality samples [207]. However, in actual scenarios, there are usually different equilibrium states in the generation of adversarial networks. If the model training fails to converge to the optimal equilibrium point, it will lead to insufficient model learning, that is, the Nash balance task encounters the problem of multiple balance points and weak correlation between them. Therefore, how to find all the equilibrium points is still one of the recognized difficulties in the current academic circles. Influenced by this restriction, there are many unfavorable factors, such as the non-optimal equilibrium state, in the theoretical level of the generative adversarial mechanism. Unfortunately, the existing methods cannot deduce the global optimal Nash equilibrium point of the model from the theoretical proof point of view, which leads to the model falling into local equilibrium and limiting the actual performance of the model. This direction therefore remains an important challenge for the future.

#### 4.1.3. Transfer Learning

##### Identifying Appropriate Source Domain

Industrial data are usually large-scale and complex. In the mass data, the valuable information related to the fault is quite limited, and different equipment, different time and different running states will lead to differences in the collected data, which is not ideal. It is difficult to find an appropriate source domain, which contains enough training examples and annotates them with sufficiently accurate label information to achieve the target task. Some specific public datasets (such as CWRU dataset) are used to verify the proposed methods, which are often very effective. However, these may be far from the actual working condition data, so it is difficult to apply to actual industrial fault diagnosis and prediction. In addition, how to better transfer the complex domain is also a research direction.

##### Negative Transfer and Transferability

Many proposed transfer learning algorithms assume that the source and target domains are interrelated in some sense. However, if the assumption does not hold, it can lead to a negative migration. Therefore, how to ensure that negative transfers do not occur is a key issue in transfer learning. Whether negative transfer will occur may depend on several factors, such as the correlation between the source domain and the target domain, and the ability of learners to discover transferable and beneficial parts of cross-domain knowledge. In order to avoid negative transfer learning, we need to first study the transferability between the source domain or task and the target domain or task. In 2014, Yosinski et al. [208] published a paper, “How transferable are features in deep neural networks?”, which used experiments to study the portability of different layers of deep neural networks, providing a very high guiding significance for DTLs. At present, the DTL model has made a breakthrough in the field of computer vision, because many studies have proved that the DL model can learn more transfer features for these tasks than traditional hand-made features [209].

But for IFD, there is no research on how the features in the DTL model can be transferred. In the industrial scene, one of the effective measures taken to improve the performance of the IFD model based on DTL is to transfer only the public knowledge that is helpful to the target learning task, while avoiding negative transfer. A related question is, when the whole domain cannot be used for transfer learning, whether we can still transfer some domains in the target domain for useful learning. Negative transfer and mobility are topics to be further studied in DTL. Therefore, there is a strong engineering demand for effectively quantifying the degree of transfer, guiding the selection of data samples, promoting the positive transfer of models and further improving the scientific nature of transfer.

##### Prior Knowledge

For IFD based on DTL, many scholars pay little attention to the prior knowledge behind the data, and it is difficult to combine the knowledge of human experts with DTL network learning through explanation. Therefore, future researchers can introduce prior knowledge into the proposed method to build a more targeted and applicable diagnostic model. In the future, research can be carried out from the following aspects: (1) using the prior knowledge of the mechanism and dynamic response of common faults in mechanical systems, we can find the corresponding relationship between network structure and fault mechanism in the research of network model theory; (2) mining the essence of data, seeking the visualization of recognition and prediction results from the data point of view.

##### Generalization Performance

In fault diagnosis, the objects we study have different working conditions, such as different maintenance histories, different fault modes or different degrees of this fault, etc., which will lead to certain influences on the generalization performance of the established model. In the future, we can look for some features of causality to enhance the generalization of the model, that is to say, further research on causality interpretability.

#### 4.1.4. Application

##### Motivation

Generally, DTL has four transmission motives in mechanical fault diagnosis. One is different working conditions, such as different rotating speeds and working loads. This motivation is the mainstream at present, and the second one concerns different types of failures, that is, there may be label differences between the source domain and the target domain. As described in Section 3.1.2, the third issue is the different positions, that is, the test data are collected by sensors in different positions of the machine. At this time, the data vibration characteristics are different, and the feature distributions of the source domain and the target domain usually do not overlap. However, these data still conform to the same machine health condition, and there are common basic characteristics among them. Therefore, it is feasible and promising to study the knowledge transfer of fault diagnosis among different sensors [210,211,212]. The fourth is different machines; Guo et al. [213] combined adversarial discriminant methods and difference-based methods, using MMD to narrow the differences between features after acquiring domain-independent features in the shared space through adversarial training, which is one of the earliest jobs of different machines.

DTL cross-machine fault diagnosis is studied in the literature [214,215,216,217]. In the past, when studying the fault diagnosis of different locations and different machines, the selected source domain has the same task as the target domain, that is, the health status classification of the source domain and the target domain is the same. However, in the actual situation, different types of faults may also appear in cross-location and cross-machine scenarios, and the fault classes unrelated to the target domain in the source domain may have a negative impact on transfer learning. This is also a situation that needs to be considered.

##### Complex Fault Diagnosis

Many effective diagnostic methods are proposed in the study of fault diagnosis methods, but there are still insufficient diagnostic methods for early faults, weak faults, compound faults, system failures and intermittent faults, and reliable diagnostic methods are limited. Damage and early failures are inevitable during system operation, which entails a weak dynamic response. Because of the coupling of multiple factors and complex transmission paths, it is often difficult to trace the causes of compound faults and system faults effectively with a single signal processing method.

There are still multi-point compound faults in rolling bearings (the bearings produce different degrees of faults at different fault positions at the same time). At present, the advanced signal processing technology is mainly used to analyze the characteristic frequency components of the monitoring signals corresponding to different fault positions, and then the fault positions can be judged. Affected by the noise interference and mutual coupling of fault components, signal processing methods are difficult to popularize and apply. Some researchers have studied the above faults; Huang et al. [218] proposed the DTL method combined with a transferable capsule network (TCN), which is used to decouple the composite faults of machinery under different operating conditions. Chen et al. [219] proposed a DATN method that takes into account data on different fault severities, compound faults, and noise contamination. However, at present, these methods still lack theoretical reference, and few researchers have studied complex fault diagnosis using multi-heterogeneous sensing data, which will also be a valuable research direction in the future.

##### Prognostic and Health Management

Prognostic and Health Management (PHM) refers to the technology of evaluating and managing the health status of equipment by using a large amount of condition monitoring data and information, and statistical algorithms or models. Residual useful life (RUL) prediction technology is one of the key technologies of PHM; studies [220,221,222,223] have used DTL to solve the RUL problem, but the existing research mostly focuses on the prediction of RUL under single working condition and single failure mode, ignoring the consideration of environmental conditions and operating conditions to a certain extent. The prediction of RUL under variable working conditions and multiple failure modes deserves further study.

According to the summary given in the literature, there are few published RUL forecasting datasets, and the data scale is not large, which also limits the further development of DTL in the field of RUL forecasting to a certain extent. We can consider using the data enhancement ability of generative DTL to solve the problem of missing RUL forecasting datasets.

### 4.2. The prospect of DTL in Fault Diagnosis

In recent years, new methods of DTL fault diagnosis have emerged, and the experimental results are more accurate. Here, this article looks forward to the future research direction in order to further strengthen the existing work.

#### 4.2.1. Establish a Standard Large Database

This is an important foundation and resource for research on big data diagnosis. The establishment of a standard big database is of strategic significance to the innovation of diagnosis technology, the revelation of the fault evolution mechanism and large-scale scientific research cooperation. At the same time, because the models used in DTL learning methods are usually complex, the performance of mechanical fault diagnosis based on DTL learning depends largely on the size and quality of datasets. At present, almost all fault diagnosis examples use CWRU. On the other hand, the depth of the DTL learning model is limited by the size of the dataset. Therefore, it is meaningful to establish a standard mechanical database.

#### 4.2.2. Combined with Fault Diagnosis Theory

Even in the context of big data, DTL is only a method for processing data, and it cannot be the key to solve the problem of fault diagnosis. The knowledge of fault diagnosis theory that has accumulated for a long time should contribute to the application of a DTL learning model in mechanical health monitoring. For example, the simple feature extraction of the data before inputting it into the network can effectively reduce the depth of the model, and the proper regularization term can improve the diagnostic accuracy of the model. While pursuing the automatic feature extraction ability of deep learning and the advantages of transfer learning in knowledge transfer, the rational use of fault diagnosis theory is a shortcut.

#### 4.2.3. Multi-Technology Fusion

Large-scale industrial systems often encounter problems such as dynamics, uncertainty, vulnerability, openness and multi-fault concurrency. If only a single fault diagnosis technology is adopted, there will be problems such as low accuracy and weak generalization ability, and it is difficult to achieve satisfactory diagnosis results. We can combine some new technologies with DTL technology to complement each other, and study the fault diagnosis method of multi-technology integration, so as to effectively improve the sensitivity, robustness and accuracy of the fault diagnosis system. This will reduce its uncertainty and help us estimate the severity of the fault while locating the source of the fault, truly combining theoretical research with practical industrial engineering applications. At present, some new technologies have been combined with DTL fault diagnosis and achieved good results.

##### Reinforcement Learning

Reinforcement learning can use specific feedback functions to determine optimal decisions by learning from examples. DTL can use models trained on other data to help with training. Combining DTL and reinforcement learning can further leverage small-scale data to train better models that cannot be achieved by other methods with the same amount of data. At present, there has been some progress made in transfer reinforcement learning [224]. How to better combine the two and apply them in the field of fault diagnosis to greatly reduce the problem of model accuracy decline caused by insufficient fault data is the focus of the next step.

##### Meta-Learning

Meta-learning is a typical model-based method, which improves the generalization ability of models under different classification tasks. Through training a small number of samples, the meta-learning method focuses on seeking fast and accurate model adaptation. The data enhancement method of the generative anti-transfer network solves the problem of data imbalance classification from the data level, and it can be solved from the algorithm mechanism level by combining with a meta-learning method in the future. In addition, meta-learning can also be combined with adversarial learning to enable adversarial unsupervised domain adaptation for cross-domain fault identification [225].

##### Graph Convolutional Network

GNN is a deep learning algorithm specially designed for graphic data, which stands out because of its efficient data relationship mining ability. It has also been successfully applied in the field of fault diagnosis to improve the accuracy and robustness of diagnosis results. Li et al. [226] and Zhang et al. [227] proposed to extract domain invariance and differentiation characteristics by using GNN to achieve domain adaptation. Most of the current work does not take prior knowledge into account when constructing diagrams, and the interpretability of GNNs has not been properly addressed.

##### Few-Shot and Zero-Shot

The distribution of data is not average, and it shows the characteristics of such a long tail distribution. Physical knowledge can be integrated into the network to reduce the size of the required training set, and a small amount of learning is devoted to learning from a limited number of examples, which is a promising method for solving the problem of cross-category fault diagnosis. Xu et al. [104] and Li et al. [105] used few-shot learning for DTL troubleshooting, but the more challenging zero-shot technique is rarely used in DTLs troubleshooting.

##### Attention Mechanism

The attention mechanism is widely used in images. In the transfer learning of images, the mobility of different areas of images is different, and the areas with low mobility will cause negative transfer in the training process. The domain adaptation method needs to focus on knowledge transfer in the areas with high task correlation in the image, while ignoring other irrelevant background information.

Wang et al. [228] introduced attention mechanisms into adversarial domain adaptation methods, enabling the network to automatically learn which parts to pay attention to during the transfer process.

Wang et al. [229] proposed a multi-domain weighted adversarial transfer network, and designed multi-domain adversarial and attention-weighted modules to consider the characteristics of the multi-mode structure and solve the influence of the local non-transitive region of the signal. The attention mechanism enhances the interpretability of the model, but it also results in slower model training.

#### 4.2.4. Fault Classification Diagnosis

The failure-based evolution process can be divided into significant failures and minor failures, and the mode characteristics of different faults are different. Small faults are faults that deviate from the normal state of the process variable to a small extent. If minor faults are not handled, they can pose a safety hazard to the operation of the entire system.

If a multi-level diagnostic framework can be designed in combination with the deep network framework structure, it will be conducive to realizing the real-time monitoring of significant faults and effectively improving the identification rate of small faults with strong concealment and randomness. In addition, different faults have different impacts on system performance, and small faults are also very likely to cause great deviations, so further research needs to be done on how to combine evaluation functions and decision rules at the decision-making end to reduce the false identification rate and realize the real-time and accurate emergency regulation of multi-variable industrial processes.

#### 4.2.5. Online Transfer Learning

At present, most of the mechanical fault diagnosis models based on DTL have been tested and verified, but these have only been conducted off-line. Both the source domain and the target domain data have been acquired, but in the real scene, the data (target domain) will be continuously input in the form of data stream. The online data not only reflect the latest changes in the current running state of the system, but also contain the cumulative correlation of the running process. At present, online DTL has been the focus of some research on self-adaptation and online feature selection in the multi-source domain and target domain, but the research work on fault diagnosis is generally less common. Online DTL, if it can be applied to the fault prediction direction of industrial field, will greatly improve the real-time stability of mechanical equipment, find problems earlier and faster, and reduce the risk of industrial accidents.

#### 4.2.6. Energy Efficiency Ratio

The method based on DTL and its improvement has achieved good application results in bearing fault diagnosis, but its complex structural parameters need intelligent optimization, which will consume a lot of calculation time and have a great influence on real-time monitoring in industrial applications. How to shorten the training time restricts the further development of intelligent fault diagnosis. In DTL fault diagnosis, researchers have added a lightweight structure to save calculation time [44]. How to improve the accuracy of the model and reduce the scale of the model is a research direction.

#### 4.2.7. Distributed Fault Diagnosis Model

With the continuity, high-speed, systematization and automation of industrial production, as well as the networking of enterprise management, large-scale key industrial equipment has been presented as a distributed and open scale system with a distinct hierarchy. The development of distributed fault diagnosis technology will provide a potential way for the design and implementation of a large-scale diagnosis system. This technology can be used to solve the intelligent diagnosis of large-scale faults, and form decentralized subsystems facing specific problems or relatively simple fault equipment. This is a method that will need further research in the future to coordinate all subsystems to conduct fault diagnosis and system reliability evaluation in parallel and cooperatively. How to establish a distributed fault diagnosis algorithm based on DTL has become a big challenge.

#### 4.2.8. Auto Machine Learning

Due to the characteristics of strong personalization and high noise of actual fault data, the traditional data analysis methods still require further exploration and in-depth study. Fortunately, the rise of automatic machine learning technology provides a feasible scheme and idea for solving problems. At present, the goal of automatic machine learning is to automate the whole process of data analysis, including automatic data acquisition and experimental design, automatic data cleaning and missing data filling, automatic feature selection and transformation, automatic model discovery, evaluation and interpretation, automatic computing resource allocation, automatic super-parameter optimization, automatic inference, and automatic model detection and anomaly detection. At present, there is still a long way to go to realize the automatic machine learning of intelligent fault diagnosis. In the future, the intelligent diagnosis method based on automatic machine learning will become one of the directions that many scholars in the field focus on and strive to break through.

#### 4.2.9. Digital Twin

At present, the research on fault diagnosis and residual service life prediction models of equipment usually ignores the complex interaction mechanism between equipment structure and physical attributes, and cannot reflect the dynamic physical entity characteristics of equipment. For complex equipment with a multi-coupling structure and multi-disciplinary technology, the difficulty of effective data collection and the complexity of the signal transmission path seriously affect the landing of health monitoring technology. At the same time, due to the lack of fault mechanism cognition and the perception limitations in a complex environment, it is difficult to fully mine the parameters related to the running performance of equipment, which limits the improvement of the accuracy of equipment health assessment. Therefore, the research on the intelligent health assessment method of digital twin-driven equipment, continuously improving the approximation between simulation model and physical reality, and promoting the interactive consistency and synchronization between physical space and network space, will have a profound impact on the development of fault prediction and health management technology.

#### 4.2.10. Others

Xu et al. [230] proposed a deep DACMD that achieves higher and more stable transfer learning diagnostic accuracy through adversarial domain classification networks and the regularization method of CMD. Wang et al. [231] proposed a new anti-domain adaptive method based on high-order moment matching, which significantly reduced the distribution difference across domains by using combined high-order moment statistics (HMS) and adversarial learning. Kuang et al. [232] proposed a new self-supervised double classifier adversarial transfer learning network that combines self-supervision and supervised optimization during training. Oh et al. [233] propose a deeply transferable motion adaptive fault detection method, which uses residual convolutional neural networks to enhance the feature extraction performance of simple motions, and adaptively detects faults on multi-axis motion through the contrast learning of opposites. Zhuang et al. [234] propose an adversarial domain generalization framework with regularized learning (ADGR) for health assessment to unearth potential domains. The proposed inter-domain regularization and semantic consistency regularization are used to constrain two-stage extractors to avoid feature drift and semantic collapse.

### 4.3. Summary

Although the fault diagnosis technology based on DTL has greatly promoted the development of fault diagnosis field, it still faces various challenges and needs more and more research. This section summarizes the current research challenges and looks forward to the future development trends of DTL. At present, the ultimate goal of different experiments and studies is to outline fault diagnosis and prediction methods that will be successful. Considering the actual complex industrial environment, the performance evaluation of the proposed diagnosis methods is generally focused on one aspect, and usually only the accuracy or efficiency is considered, but the cost, stability, generalization ability and other factors are not comprehensively considered. There is still a long way to go before DTL is applied in practical engineering. To summarize, this section provides an in-depth analysis of the current hurdles impeding the widespread application of DTL in IFD, and lays out a roadmap of potential future developments that could revolutionize the field.

## 5. Conclusions

This review presents a comprehensive examination of ADTL within the field of IFD. It initially introduces the fundamental aspects of DTL, followed by a thorough summary of recent advancements in ADTL fault diagnosis. ADTL, emerging as a vital concept, is classified into non-generative and generative paradigms based on the presence or absence of sample generation. The paper scrutinizes their applications within the sphere of IFD, providing significant insights into the capabilities of these models. Following the discussion of ADTL, the review encapsulates the current challenges in employing DTL for large-scale mechanical diagnosis. These include critical issues such as data imbalance, negative transfer, and adversarial training stability. It further identifies future research directions, projecting trends in DTL within the fault diagnosis domain, and offers constructive recommendations to address these existing challenges. Conclusively, the review emphasizes the immense potential of ADTL, providing a valuable guide for its optimization and deployment in real-world industrial applications.

## Figures and Tables

**Table 1:** Common algorithms used for data distribution.

Application Scenarios	Categorization	References	Common Algorithms Used
Varying Working Conditions	DANN	Jiao et al. [35], Jin et al. [36], Mao et al. [38], Mao et al. [39], Wang et al. [40]	DL-ADAN, DDA-RNN, DTDA, MMD + DANN, DATTCN
	JDA	Jiao et al. [47], Zhao et al. [48] Li et al. [49]	RJAAN, IJMMD + Adversarial domain adaptation, AJDA
	DAAN	Jiao et al. [55], Tian et al. [56], Xu et al. [57], Wei et al. [58], Zhao et al. [59],	MAAN, SAAN-EAS, VMD-EE + TL, DTAL, MSANA
	C-MMD	Lee et al. [66], Shao et al. [67], Jia et al. [68], Zhang et al. [69], Shao et al. [70], Liu et al. [71], Li et al. [72], Li et al. [73], Li et al. [74], Wan et al. [76]	AIIDA, MK-MMD, DGDAN, SCDA + LMMD, DCMADA, ADA-MMA
	C-WD	Liao et al. [77], Li et al. [78], He et al. [79], Li et al. [80], Zhang et al. [82], Bao et al. [83], Zhang et al. [84], Wang et al. [86], She et al. [87], Wang et al. [88], Zou et al. [89], Jia et al. [90], Zou et al. [91], Han et al. [92], Liu et al. [93], Wang et al. [94], Xu et al. [95]	DSDGN, C-ASSF, WGAN + minimum singular value, MAAN, WDMAN, TLADA, WDDMA, WACCVAE, DCWANs, HDAN, FCWAN, DADAN, DAN-DAM
	C-CORAL	Qin et al. [97], Li et al. [98], Li et al. [99], Zhang et al. [100]	PSADAN, DAACA, ADA-AMCA, eDANN
Across Different Machines	DANN	Wang et al. [41], Zhu et al. [42]	DANN, Standardize datasets + DANN
Others (Insufficient label sample, Noise label, etc.)	DANN	Mao et al. [37], Mao et al. [43], Wu et al. [44], Di et al. [45]	SDANN, LDANN, DANN, Joint training (DANN),
	JDA	Yang et al. [50], Zhang et al. [51]	CDAN + JDA, SNMCAN
	DAAN	Fan et al. [60]	DWQDAN
	C-MMD	Zhou et al. [75]	Res-BPNN + MK-MMD,
	C-WD	Xiang et al. [81], Cheng et al. [85], Ying et al. [96]	WDATL, WD-DTL, WAADA

**Table 2:** Common algorithms used for incompletion sets.

Categorization	References	Method
Small sample	Han et al. [101], Li et al. [102], Wu et al. [103], Xu et al. [104], Li et al. [105], Wang et al. [106], Han et al. [107],	DACNN, DA-PTL, TMCD, CFDM, DATCNN, C-WGAN
Class imbalance	Guo et al. [108], Yang et al. [109], Wu et al. [110], Kuang et al. [111], Tan et al. [112], Xia et al. [113]	Two-stage training strategy, DPTL, deep Imba-DA, CIATL, MiDAN, DPADA

**Table 3:** Common algorithms used of inconsistent label space.

Categorization	References	Method
Partial set	Wang et al. [114], Liu et al. [115], Li et al. [116], Jiao et al. [117], Li et al. [118], Zhao et al. [119], Wang et al. [120], Hao et al. [121], Deng et al. [122], Mao et al. [123], Qian et al. [124]	Unilateral alignment, SPADA, Class-weighted, MWDAN, WANT, DA-GAN, MDWAN, DCs + SRPS, Balanced center alignment and weighted adversarial alignment, PT-ELF, MWSAN,
Open set	Zhang et al. [126], Zhao et al. [127], Zhu et al. [128], Li et al. [129], Li et al. [130], Li et al. [131], Li et al. [132]	Instance-level weighted, Dual adversarial network, ANMAC, Global–local dynamic adversarial network, SAE, DATLN, TSTAN
Universal	Chen et al. [133], Yu et al. [134], Zhang et al. [135], Li et al. [136]	TWUAN, BWAN, Additional outlier identifier, ADGN

**Table 4:** Common algorithms used for complex domain.

Categorization		References	Common Algorithms Used
SSMT		Li et al. [137], Deng et al. [138], Ragab et al. [139]	AMDA, CRCAA,
MSST	Multi-domain adaptation	Wei et al. [141], Xu et al. [142], Si et al. [143], Chai et al. [144], Rezaeianjouybari et al. [145], Huang et al. [146], Zhu et al. [147], Zhang et al. [148], Feng et al. [149], Li et al. [150], Chai et al. [151], Li et al. [152]	IFDS, MSDA, MADN, FTD-MSDA, FTD-MSDA, MDAAN, ADACL, MDA, MRTN
	Domain generalization	Chen et al. [153], Han et al. [154], Zhang et al. [155], Huang et al. [156]	ADIG, IEDGNet, DACN

**Table 5:** Generative adversarial adaptation model.

Categorization	References
Direct extended data	Li et al. [157], Guo et al. [162], Shi et al. [163], Wu et al. [164], Peng et al. [165], Li et al. [166], Zhu et al. [167], Xie et al. [168], Wang et al. [169], Jiao et al. [170], Zhao et al. [171], Liu et al. [172], Zhu et al. [173], Li et al. [174]
Combined data extended with transfer learning	Sankaranarayanan et al. [175], Xu et al. [176]

## Data Availability

Not applicable.
